# Recent Developments in Diagnosis of Epilepsy: Scope of MicroRNA and Technological Advancements

**DOI:** 10.3390/biology10111097

**Published:** 2021-10-25

**Authors:** Ritam Bandopadhyay, Tanveer Singh, Mohammed M. Ghoneim, Sultan Alshehri, Efthalia Angelopoulou, Yam Nath Paudel, Christina Piperi, Javed Ahmad, Nabil A. Alhakamy, Mohamed A. Alfaleh, Awanish Mishra

**Affiliations:** 1 Department of Pharmacology, School of Pharmaceutical Sciences, Lovely Professional University, Phagwara 144411, Punjab, India; ritambanerjee02@gmail.com; 2 Department of Neuroscience and Experimental Therapeutics, College of Medicine, Texas A&M University Health Science Center, Bryan, TX 77807, USA; tanveersingh@tamu.edu; 3 Department of Pharmacy Practice, College of Pharmacy, AlMaarefa University, Ad Diriyah 13713, Saudi Arabia; mghoneim@mcst.edu.sa; 4 Department of Pharmaceutics, College of Pharmacy, King Saud University, Riyadh 11451, Saudi Arabia; salshehri1@ksu.edu.sa; 5 Department of Biological Chemistry, Medical School, National and Kapodistrian University of Athens, 11527 Athens, Greece; angelthal@med.uoa.gr (E.A.); cpiperi@med.uoa.gr (C.P.); 6 Neuropharmacology Research Strength, Jeffrey Cheah School of Medicine and Health Sciences, Monash University Malaysia, Bandar Sunway, Subang Jaya 47500, Selangor, Malaysia; yam.paudel@monash.edu; 7 Department of Pharmaceutics, College of Pharmacy, Najran University, Najran 11001, Saudi Arabia; jaahmed@nu.edu.sa; 8 Department of Pharmaceutics, Faculty of Pharmacy, King Abdulaziz University, Jeddah 21589, Saudi Arabia; nalhakamy@kau.edu.sa (N.A.A.); maalfaleh@kau.edu.sa (M.A.A.); 9 Vaccines and Immunotherapy Unit, King Fahd Medical Research Center, King Abdulaziz University, Jeddah 21589, Saudi Arabia; 10 Department of Pharmacology and Toxicology, National Institute of Pharmaceutical Education and Research (NIPER)—Guwahati, Changsari, Guwahati 781101, Assam, India

**Keywords:** epilepsy, diagnosis, EEG, CT, MRI, PET, SPECT

## Abstract

**Simple Summary:**

Through epilepsy is one of the leading causes of poor mental health, increased school dropout, and socioeconomic burden, yet the diagnosis remains a challenging concern especially in developing countries. There has been a significant advancement in the diagnosis of different epileptic conditions. Therefore, the present work is focused on analyzing the technological advancement and scope of microRNA in the development of effective diagnostic tools and developing effective clinical management of epilepsy.

**Abstract:**

Epilepsy is one of the most common neurological disorders, characterized by recurrent seizures, resulting from abnormally synchronized episodic neuronal discharges. Around 70 million people worldwide are suffering from epilepsy. The available antiepileptic medications are capable of controlling seizures in around 60–70% of patients, while the rest remain refractory. Poor seizure control is often associated with neuro-psychiatric comorbidities, mainly including memory impairment, depression, psychosis, neurodegeneration, motor impairment, neuroendocrine dysfunction, etc., resulting in poor prognosis. Effective treatment relies on early and correct detection of epileptic foci. Although there are currently a few well-established diagnostic techniques for epilepsy, they lack accuracy and cannot be applied to patients who are unsupportive or harbor metallic implants. Since a single test result from one of these techniques does not provide complete information about the epileptic foci, it is necessary to develop novel diagnostic tools. Herein, we provide a comprehensive overview of the current diagnostic tools of epilepsy, including electroencephalography (EEG) as well as structural and functional neuroimaging. We further discuss recent trends and advances in the diagnosis of epilepsy that will enable more effective diagnosis and clinical management of patients.

## 1. Introduction

Epilepsy is a common neurological disorder, characterized by the tendency of recurrent seizures, which may take a variety of forms and result in abnormally synchronized neuronal discharges. Seizure types depend on the specific brain circuit being affected. Epilepsy affects 0.5–1% of the population, corresponding approximately to 70 million people worldwide. It may be genetic in origin (idiopathic) or may develop after brain damage, such as trauma, stroke, infection, tumor growth, or other structural or metabolic causes [1,2,3,4,5].

Epilepsy treatment is highly demanded since the continuous abnormal neuronal discharges and subsequent recurrent seizures may cause damage to various brain regions, leading to the development of several neurological or other disorders, such as neurodegeneration, motor impairment, abnormal hormone release (such as ACTH, prolactin, FSH, TSH, etc.), psychosis, etc. [6]. Hence, prompt, and proper diagnosis of epilepsy is essential for effective treatment. Traditional diagnostic methods include electroencephalography (EEG), structural and functional neuroimaging (CT scan, MRI, PET, SPECT, etc.), and blood tests for the detection of the abnormal electrical activity of the brain or the potential identification of specific serum biomarkers. Although these methods are very helpful in detecting abnormal electrical discharges in the brain or identifying potential causes of epilepsy, they might lead to false positive or false negative results [7]. Epilepsy can be difficult to be diagnosed, even for experienced clinicians. Upon clinical suspicion, the gold standard diagnostic method of epilepsy is EEG, which can detect abnormal electrical discharges in the brain. However, these abnormalities may or may not be associated with epilepsy. Some other conditions, such as infections, CNS medications (psychotic drugs such as antidepressants, antipsychotic, antiparkinsonian, etc.) as well as trauma and excessive stress can affect the results of EEG. Additionally, non-epileptic seizures (NES) due to psychological and physiological paroxysmal events, such as epileptic manifestations, can considerably complicate diagnosis [8].

The detection of serum biomarkers is not a reliable strategy since they may be associated with other non-epileptic medical conditions, raising concerns about their specificity. For instance, the levels of antidiuretic hormone, which is a biomarker of hypothalamic activity, may be reduced or elevated in response to drugs or alcohol and may increase in response to emotional and physical exercise or pain [9]. Hence, this hypothalamic hormone cannot be used as a specific serum biomarker. In addition, prolactin which is secreted from the hypothalamus and is sometimes used as a biomarker of epilepsy can be often related to other conditions, such as milk production, breast development, and pituitary tumors [9].

Poor diagnosis often results in inappropriate pharmacotherapy leading to high morbidity of patients with epilepsy. Herein, we summarize diverse approaches to the diagnosis of epilepsy and discuss recent advances in this field, which may facilitate prompt and accurate diagnosis, leading to a more effective clinical management.

## 2. Diagnostic Techniques

**Figure 1:**
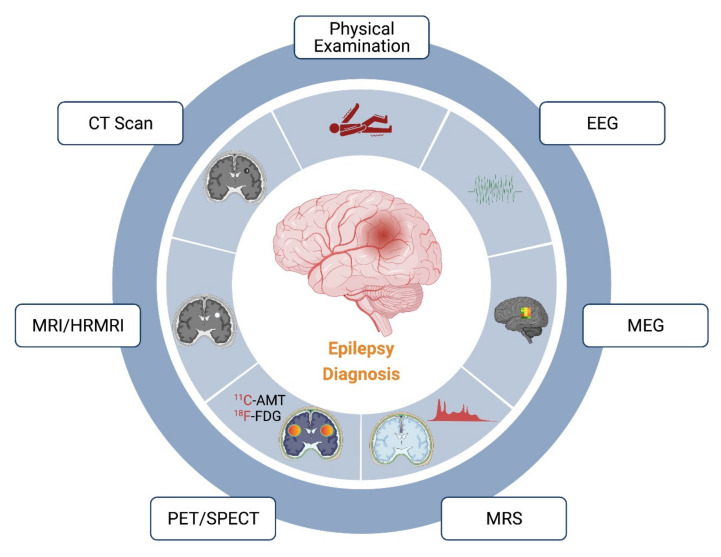
Current methods and techniques available for the diagnosis of epilepsy. The main diagnosis of epilepsy relies majorly in the physical examination followed by EEG analysis. However, the identification of the epileptic locus might be ambiguous with these methods. Therefore, the use of more elaborative techniques, such as CT, MRI/HRMRI, PET/SPECT, MRS, and MEG, is recommended for demarcation of the epileptic foci.

Epilepsy in children remains poorly diagnosed and contributes to school dropout, mental retardation, and poor quality of life. The current diagnostic pattern mostly relies on the conventional EEG recording. Antiepileptics provide satisfactory control only in up to 60% of patients with epilepsy, with the rest remaining refractory. In these refractory epileptic cases, surgical removal of lesions remains a putative approach for satisfactory seizure control. For ablation surgery, the success depends on the extent of effective demarcation of the epileptic lesion area. In these cases, the use of advanced diagnostic techniques or a combination of other diagnostic tools, such as CT, MRI, PET/SPECT, MEG, MRS, etc., is required. Different diagnostic tools for epilepsy have been illustrated in Figure 1 and discussed herein.

### 2.1. Electroencephalogram (EEG)

EEG measures abnormalities in the brain’s electrical activity or the dynamics of the brainwaves. The cerebral cortex of the human brain consists of two hemispheres, and each one of them has four lobes: frontal, temporal, parietal, and occipital. In most clinical applications, during EEG, 19 electrodes (plus system reference and ground) are usually used to detect and record the electrical discharge activities in the brain. There are two types of electrodes, including surface and deep ones. The surface or scalp electrodes are affixed to the surface of the scalp and can detect the discharge patterns originating at the superficial region of the cerebral cortex. They can be unipolar (where the active terminal is placed on the cerebral cortex while the indifferent terminal is placed at another part of the body away from the cortex) or bipolar (where the two terminals are placed on different parts of the cortex). The deep electrodes are inserted under the scalp for recording the electrical activity in subcortical brain regions [10].

For EEG recording, the electrodes are placed on different parts of the brain and the activation of a bunch of electrodes reflects the activation of the cortex part that is proximal to these specific electrodes [10]. EEG can confirm a diagnosis, and can also clarify the type of epilepsy, i.e., when only a certain part or a hemisphere is affected, then it denotes a partial seizure, whereas when the whole cerebral cortex is affected then it denotes a generalized seizure. The electrodes with odd numbering are placed in the right hemisphere, while the electrodes with even numbering are placed in the left hemisphere. The CZ and PZ electrodes (ground or common reference points) are placed in the median line [10]. Additionally, in the cases of refractory or drug-resistant epilepsy, the part of the cortex affected can be detected and the surgical removal facilitated. In EEG, four types of waves are studied: “beta” waves (15–60 Hz); “Alpha” waves (8–12 Hz); “Theta” waves (6–7 Hz); and “Delta” waves (1–5 Hz) [11]. In normal physiological conditions, the amplitude and frequencies of these waves are known. In the case of epilepsy, EEG obtained during the asymptomatic period may show an abnormal wave pattern, including spikes, or spikes followed by slow-wave and poly-spikes, among others. These abnormalities are known as interictal epileptiform discharges (IEDs) [12].

There are some characteristic features of the paroxysmal discharge patterns in epileptic patients; the discharges have an abrupt onset, with a progressive increase in amplitude and frequency, followed by isoelectric EEG. These abnormal discharges occur for a short period, usually 5–10 s [12]. The appearance of median sharp waves of high frequency (they are in the normal EEG pattern and not in the paroxysmal discharge pattern) for 1–2 s under normal conditions, does not indicate epilepsy but rather a higher future risk of epilepsy (Leach et al., 2006). There are different protocols of EEG measurement, including routine (standard) EEG (20–30 min), prolonged EEG (1–2 h), long-term monitoring (24 h–3 days), ambulatory EEG, and video-EEG monitoring.

Along with the data obtained from these EEG monitoring methods, the medical history of the patient is of great importance to differentiate NES from ES. There are various indications for EEG, including the clinical doubt of epilepsy or seizure, reconsideration of the preliminary diagnosis of epilepsy, categorization of an epileptic patient to different epilepsy types, variations in seizure patterns, SE (status epilepticus) monitoring, seizure frequency monitoring, pathophysiology of NES, loss or disturbance of awareness, encephalopathy, encephalitis, dementia, cerebral vascular disease, paroxysmal behavior variations, psychic or supplementary behavioral changes, coma, brain death, genetic disorders, metabolic disorders, regression, etc. [13].

Electrocorticography (ECoG) is basically an invasive procedure, in which the measurement of electrical activities of the cortex is performed. First, the skull over the interested area of cortex is surgically removed and then electrodes are placed directly over the bare surface of the cortex, followed by the measurement of the electrical activities. It consists of two types, i.e., intraoperative ECoG and extraoperative ECoG. Insertion of ECoG for the management and diagnostic purpose of epilepsy has proven to be advantageous. The use of ECoG has depicted that verbal insufficiency in epileptic patients was associated with neuronal activation in the spatiotemporal region of the brain [14]. Although, there is a considerable progress in the implementation of non-invasive systems for the localization of epileptic foci, in a few cases the localization is not conclusive enough to identify the origin of epileptic discharges. In these cases, ECoG is utilized to direct the surgical removal of epileptic abrasions, in order to achieve a better surgical outcome. Anesthesiologists also make use of the ECoG techniques for the selection of suitable anesthetics for epileptic surgery [15]. Successful accomplishment of the surgical removal of the seizure-affected region of the brain relies on the appropriate identification of the seizure loci and on the comprehensive amputation of epileptic abrasion. Both can be achieved effectively after implementing intraoperative ECoG. In another study, it was observed that epileptic patients that were undergoing intraoperative ECoG-guided epileptic surgery demonstrated an improved post-surgical outcome, compared to patients getting normal or non-guided epileptic surgery [16]. Extraoperative ECoG can also be very useful in non-surgical epileptic treatments to identify the epileptic foci and to track the disease progression.

Advantages: This is a very accurate, relatively low-cost, non-invasive (in the case of scalp EEG) method of diagnosing epilepsy, as the epileptic abnormal paroxysmal discharges are easily detectable with a high temporal resolution. Many neurological disorders can be evaluated by this method, enabling the identification of epilepsy type, seizure circuitry, and appropriate treatment. It can further allow the quantification of the frequency of seizures in a patient. EEG is also useful in drug-resistant epilepsy for the surgical removal of the affected part of the cortex, while it can also help differentiate NES from ES, and thus reduce the occurrence of misdiagnosis.

Disadvantages: Although there are several indications, as mentioned above, the EEG reading by itself may be inadequate for epilepsy diagnosis. However, misdiagnosis can occur when the discharge pattern is not well understood. It also provides poor spatial resolution, since it can only grossly identify the part of the cortex being affected. EEG cannot effectively record neuronal activity in the deeper layers of the brain cortex.

### 2.2. Computed Tomography (CT SCAN)

A CT scan is a type of radiological imaging technique that produces a detailed 2D image. The Greek meaning of the word “tomo” is section, so tomography means sectional radiology. Computer tomography is a special type of radiological imaging technique in which a computer is used for the mathematical reconstruction of a tomographic plane or slice. It is also known as computerized axial tomography. Generally, the scans are obtained in an axial or transverse plane, and via a computer they are converted into coronal and sagittal planes to get a 3D image from a 2D CT scan image. A contrast material is often used for better visualization, appearing white (hyperdense) in the image. The epileptic foci in the cortex can be initially identified via EEG, and then structural deformities of that part of the brain can be visualized via a CT scan. Basically, CT scans reconstruct the internal structure of an object by superimposing the images obtained from the multiple projections [17]. In the CT scan, an x-ray is applied to the brain from different angles and the collected images are combined to produce a cross section image or slices taking the attenuation coefficient (µ) as a base.

The CT has the benefit of being available in most hospitals worldwide, with a low operational expense. Moreover, CT can more easily be performed in unbalanced and critically ill patients than MRI. The CT scan can diagnose most of the tumors (apart from some low-grade ones), along with substantial AV (arteriovenous) deformities, neuronal abnormalities, stroke, and infectious wounds [18]. The CT is more responsive for the discovery of calcified lesions and bone lesions, compared to MRI, but less sensitive for little cortical abrasions along with predominantly abrasions at the base of the skull, orbitofrontal and medial temporal areas [18]. The general effectiveness of CT in distinguishing lesions in focal epilepsy is less, around 30%. Thus, CT is a promising diagnostic technique for newly originated seizures in an urgent situation, but it will not be able to substitute MRI for epileptic studies.

Advantages: Painless, non-invasive, and accurate method. Short study time (15–20 min) with a high-quality image. Bones, soft tissues, and blood vessels can be imaged at the same time.

Disadvantages: Radiation exposure is a hazard and can be harmful to the fetus. Contrast materials can cause allergic reactions and renal impairment in some individuals. Furthermore, when it comes to quick diagnosis, CT scanning is ideal but detailed analysis requires MRI.

### 2.3. Magnetic Resonance Imaging (MRI)

MRI is a neuroimaging method used in radioscopy to depict the detailed structure of several body parts. It uses strong magnetic fields, magnetic field gradients and radio waves for the creation of pictures of high resolution. This method does not involve X-rays such as CT or PET scans. During the examination, the patient is placed inside of an MRI scanner containing a large magnet, and radio waves are applied from all directions, resulting in the production of a 3D image.

The basic hypothesis of MRI relies on the fact that a certain atomic nucleus can absorb some of the radio’s energy when placed in an external magnetic field; the flexible effect of spin polarization can generate an RF signal on a radio frequency coil and be detected. In clinical and research MRI, hydrogen atoms are widely used to produce several microscopic polarizations obtained by the antennas near the test subject. Most hydrogen atoms contain a lot of fat and water molecules. By changing the parameters of the pulse sequence, the differences between the tissues can be compared depending on the relaxing characteristics of the hydrogen atoms [19].

There are basically two types of weighted images in MRI, T1 and T2. T1 refers to a spin–lattice interaction, i.e., magnetization occurs in the same way as the static magnetic field, while T2 refers to a spin–spin interaction, i.e., transverse magnetic field breaks down into static magnetic forces. T1 is useful in assessing the cerebral cortex, fatty tissue, and the overall anatomy of the examined portion. T2 is useful in identifying edema, inflammation and white matter lesions [20,21]. These T1 and T2 signals can be high/bright or low/dark.

Dark signal in a T1 image may be due to increased water in the tissue, such as edema, tumor, inflammation, hemorrhage (hyperacute or chronic), and flow void. A bright T1 signal can be due to fat, subacute hemorrhage, melanin, slow blood flow, calcification, and cerebral infarction [17,18]. A bright T2 signal can be obtained due to increased water content in the tissue, edema, tumor, infarction, inflammation, subdural collection, and methemoglobin in subacute hemorrhage. A dark T2 signal is obtained due to low protein density, calcification, fibrous tissue, protein-rich fluid, and flow void [20,21].

MRI is preferred over CT procedure for the detection of posterior cranial fossa, which contains the brainstem and cerebellum. The MRI is the best choice for a neurological diagnosis [22]. Functional MRI (fMRI) may also depict the response of the brain to different stimuli, allowing researchers to learn both the structural and the functional abnormalities of epilepsy [23]. MRI is also used in the shepherded surgical procedure of epilepsy [24]. MRI can also be used for the diagnosis of temporal lobe epilepsy, neocortical lesions, multiple sclerosis, focal corticoid dysplasia, tuberous sclerosis, and Sturge–Weber syndrome [1,25,26].

Advantages: It is an advanced and accurate method. The images obtained are of very high quality, allowing proper identification and diagnosis of the cause of epilepsy. MRI also helps to determine the blood flow rate along with the white and gray matter distribution in the brain. It enables decision making for the surgical removal of the part of the cortex affected in the case of refractory or drug-resistant epilepsy while there are no radiation exposure hazards.

Disadvantages: It is an expensive procedure, and it requires very sophisticated instruments and a well-trained operator. Although it is not a painful or invasive procedure, the patients must remain still in a closed space, which can be uncomfortable. MRI cannot always distinguish between malignant and benign tumors. It cannot be used in patients with pacemakers or harboring a metallic object inside their body.

### 2.4. Positron Emission Tomography (PET)

A position emission tomography (PET) scan is an imaging technique that helps to reveal the function of tissues and organs. It is an effective way to examine the chemical activity in the brain. A PET scan uses a radioactive tracer element that appears as a bright spot reflecting chemical activity. The tracer element may be injected, swallowed, or inhaled, and then traced inside the body by radio imaging. For labeling, radioisotopes are used to detect disease before it becomes visible in other imaging tests. The PET scan was developed in the 1970s [27]. It commonly employs the radiotracer 2-deoxy-2(^18^F) Fluro-D-glucose(^18^F-FDG), which maps glucose uptake and metabolism in the brain, known as FDG-PET. The PET provides virtually unlimited possibilities regarding the study and diagnosis of epilepsy since it can trace large numbers of molecules. Its applications include the investigation of receptor–ligand/protein–protein interactions, enzymatic activities, gene expression studies, and cell and gene therapy [28].

Positron emission tomography with ^18^F-FDG integrated with computed tomography is used to effectively identify the epileptic foci. In this case, a fluorine-18 labeled D-glucose analog is used to produce the PET image and then a CT scan can be performed. By using advanced software, PET and CT scan images can be integrated to produce a high-quality image that gives both structural as well as functional information of a brain lesion or damage [29]. It can demonstrate both the metabolic and structural abnormalities of the lesion and it can be used in the diagnosis of epilepsy types induced by brain damage or shock [29].

The PET has great potential for the in vivo investigation of neurological changes that occur during epilepsy. Its application is still under validation phase [30,31]. In most cases, the ^18^F-FDG-PET is used for diagnostic purposes, evaluating metabolic changes that occur during seizures. The FDG is taken up by cells in a very similar manner as that of glucose; however, after phosphorylation by hexokinase, the FDG is not metabolized further, but rather gets trapped inside the cell. This provides a glucose utilization pattern in the brain. FDG uptake is increased in several parts of the brain during seizures [32]. Since there is elevated brain activity during seizures, more glucose is required. Therefore, the investigation of glucose uptake by different brain regions, along with MRI imaging, aids in the identification of an epileptic focus in the brain.

The PET can also identify changes in receptor expression in cases of epilepsy. By using FMZ-PET (flumazenil-PET), the activity of the GABA/CBZ receptor complex, which is a well-known receptor contributing to epileptogenesis, can be determined [33]. The PET using (^18^F) 2’-Methoxyphenyl-(N-2’-pyridinyl)-p-18F-fluoro-benzamidoethylpiperazine (MPPF) has also revealed that the bioavailability of 5-HT1A receptors in the epileptogenic zone is decreased [34].

However, PET images are difficult to interpret, as they contain poor structural details. Thus, PET analysis should be accompanied with MRI brain imaging. A frequently used technique contains the co-registering of MRI and PET images for the association of structural and functional data. The accuracy and dependability of the co-registering method critically disturb the accuracy of the results and therefore need to be properly validated. Currently, the development of tracers that would allow for the concurrent procurement of PET and MRI images is in progress [35,36,37].

Advantages: It is a highly accurate technique with many applications in the field of gene expression, gene mapping, receptor interaction, and receptor pharmacology. Detailed images of the function of different brain parts can be obtained and the acquisition of data is very fast and dynamic, while it can detect pico- and femtomolar concentrations of ligands [38].

Disadvantages: Radiolabeled probes are used to detect brain functions, imposing a risk of radiation hazards. Some probes are trapped inside the cell after metabolism [28]. PET images often capture poor analytical details and thus integration of MRI is needed. It can lead to misdiagnosis in the case of metabolic disorders and results can be questionable since there is a large number of individual variations.

### 2.5. Single Photon Emission Tomography (SPECT)

SPECT is a special type of PET test where metabolic uptake, along with blood flow, is monitored in a specific part of the brain. SPECT is a well-recognized method for evaluating neuronal activities in epilepsy through the quantification of regional cerebral blood flow [39]. The SPECT is used to evaluate the pathology when the neurological symptoms are not detectable by structural neuroimaging findings.

In this technique, a radiological tracer is administered intravenously. Through blood circulation, these elements can enter the brain cells due to their hydrophobic nature and remain there because of their conversion into hydrophilic scaffolds. However, in the case of brain disorders, the hydrophilic and lipophilic balance is disturbed. In this way, proper distribution of these tracers is not possible. The areas of improper distribution are identified as epileptic foci. Technetium-99m-hexamethyl propylene amine oxime (99mTc-HMPAO) and technetium-99m-ethylcysteinate dimer (99mTc-ECD) are used as tracers in SPECT [40,41]. The 99mTc-HMPAO reflects the blood flow arrival to specific cerebral regions and the 99mTc-ECD reflects the cellular metabolism uptake [42]. The higher gray matter concentrations, compared to white matter, contribute to better image quality. The retention of these two tracers is not linear with rCBF into the brain cells because of the initial back diffusion [43]. The high blood flow may be underrated, and the low blood flow may be overemphasized in the case of these two tracers [44]. The SPECT has applications in the diagnosis of ADHD, OCD, schizophrenia, anxiety and depression, epilepsy, neurodegenerative disorders, movement disorders, traumatic brain injury, dementia, cerebrovascular disease, brain tumors, and brain infections [45].

Advantages: This is a very sophisticated and accurate method. It provides detailed and high-resolution images. Quick determination is possible.

Disadvantages: It is a very expensive procedure; radiation hazards are possible, and a proper diet should be maintained to obtain high-quality images.

## 3. Recent Developments in PET Radiotracers

There are a lot of recent developments in the diagnosis of epilepsy, including various developments of PET, high-resolution MRI (HRMRI), diffusion tensor imaging (DTI), magnetic resonance spectroscopy (MRS), magnetoencephalography (MEG), and the development of various data analytical techniques.

### 3.1. Translocator Protein (TSPO)—PET

The radioligand of the so-called translocator protein (mostly benzodiazepine receptors) can also be used for PET analysis. The BDZ receptors, i.e., GABA receptors, are of great importance in the pathophysiology of epilepsy. A radiolabeled ligand is used that binds to the BDZ receptor, and its activity is monitored. The TSPO, which is 18 kDa in size, is situated on the outer side of the mitochondrial membrane in the central and peripheral nervous system cells. It binds with cholesterol and various kinds of drugs. High TSPO expression is found in macrophages, lymphocytes, neutrophils, and activated microglia cells [46]. The amount of TSPO is very little in the normal human brain, but increases in epilepsy due to microglia activation [47]. Activated microglia cells are associated with many CNS disorders, including epilepsy [48]. TSPO elevation is easily detected by TSPO-PET analysis [49] and can be used to visualize activated microglia cells and to a lesser extent astrocyte [50]. The newer generations of ligands are ^18^F-GE180 and ^18^F-PBR111. The ^18^F-GE180 is used to reveal the neuroinflammation duration and helps to identify neuronal abnormalities involved in epilepsy [51]. The ^18^F-PBR111 is used to predict the frequency of later spontaneous recurrent seizures [52]. However, given the fact that neuroinflammation may accompany many neurological processes, caution is needed regarding the specificity of this technique

### 3.2. Flumazenil PET

Flumazeline is a GABA_A_ receptor ligand, also known as ^11^C-flumazeline, which is vigorously used for ectopic focus determination [53]. The GABA receptor plays an important role in the pathology of epilepsy. The radiolabeled flumazenil molecule gets attached to the GABA-A receptor, detecting its abnormalities very easily [54]. Proper visualization of the hippocampus is possible due to this modification in PET [55]. Another ligand that is used for the visualization of NMDA receptors is ^18^F-GE179. Along with FMZ-PET, it enables the proper assessment of the role of the neurotransmitter system in epileptogenesis and chronic epilepsy [56].

### 3.3. ^11^C-Verapamil PET

One-third of epileptic patients are resistant to AEDs because of a specialized transporter protein called p-glycoprotein. In some cases, the intracellular concentration of AEDs can be limited by the transporter protein that pumps them out of the cell, leading to drug resistance [57]. Increased expression of p-glycoprotein is noticed in epilepsy [58]. The ^11^C-Verapamil is a radiolabeled substrate for p-glycoprotein and its uptake in the brain is correlated to the activity of p-glycoprotein. There is a uniform distribution of p-glycoprotein activity in healthy patients [59]. However, by evaluating the p-glycoprotein activity in patients with epilepsy, we could identify the epileptic center. This method can be specifically useful in refractory or drug-resistant epilepsy.

### 3.4. ^11^C-α-Methyl-L-Tryptophan (AMT) PET

The uptake of the radiolabel ^11^C-AMT is increased in epilepsy and associated with increased serotonin synthesis [60]. Serotonin plays a central role in epilepsy and the regulation of p-glycoprotein activity. Increased tracer requisite was constantly proved in tubers and correlated with ictal EEG findings [61].

### 3.5. 5-HT_1A_ Receptor Ligands and Serotonin Transporter(5-HTT)-Based PET

The ^18^F MPPF is a selective antagonist of 5-HT_1A_, exhibiting affinity towards serotonin. Hence, it can sensitively reflect serotonin variations inside the body. The ^11^C-WAY100635 and ^18^F-FCWAY are two highly potent agonists of the 5-HT_1A_ receptor [62]. They do not compete with endogenous serotonin, and therefore are devoid of any kind of interactions. Decreased expression of 5-HT_1A_ receptors in the seizure focus is a consistent finding in temporal epilepsy (TLE) studies [63,64]. These effects are marked in the hippocampus and areas involved in seizure generation [64,65]. Therefore, this 5-HT_1A_ receptor PET could be very useful in presurgical epilepsy evaluation for the detection of the seizure’s place of origin.

The ^11^C-DASB can measure the 5-HTT availability, which plays a major role in the termination of the synaptic serotonin effect [66]. During epilepsy 5-HTT expression is reduced. Thus, by measuring the reduction in 5-HTT, the epileptic foci might be effectively detected.

### 3.6. Dopamine-Based PET

The role of dopamine in epilepsy is complex and unresolved. Dopaminergic neurons in the striatum and substantia nigra play a crucial role in the termination of seizures [67]. Various types of ligands can be used for the assessment of dopaminergic pathway involvement in epilepsy, such as the presynaptic dopaminergic deficit using (^18^F)-fluoro-L-DOPA [68], decreased D2/D3- receptor binding using ^18^F-fallypride [69], decreased D1 receptor binding using ^11^C-SCH23390 [70] and reduced dopamine transporter activity using^11^C-PE2I [71]. In these cases, decreased uptake of these tracers can be observed in the epileptogenic zone of the patients with TLE [69]. These methods are used to diagnose a wide-range of epilepsy forms, including TLE, juvenile myoclonic epilepsy, idiopathic generalized epilepsy, autosomal nighttime frontal lobe epilepsy (ADNFLE), and ring chromosome 20 syndrome [69].

### 3.7. Cannabinoid-Based PET

Most of the PET ligands developed in this group have been focused on cannabinoid receptor 1 (CB1). CB1 exhibits an anticonvulsant phenomenon by modulation of neuroprotective mechanisms [72]. The downregulation of CB1 during epileptogenesis impairs this effect and promotes seizures [73]. The radioligand used for the detection of CB1 activity is 18F-MK-9470 [73]. There is an increase in the uptake at the ipsilateral temporal lobe and a decrease at the bilateral insula [74].

Along with the above analysis, radioligand labeled analysis may also aid in the identification of the effect of opioids and acetylcholine. For opioids, μ, δ, and κ receptors are examined with a radiolabeled ligand, while acetylcholine nicotinic Ach receptors (nAChR) are evaluated with a radioligand named ^18^F-fluoro-A-85380. In ADNFLE, epilepsy occurs during sleep due to the activation of the α4β2 subtype of nAChR [75]. Therefore, by increasing the uptake of ^18^F-fluoro-A-85380 (potent agonist at α4β2), the activation of α4β2 is determined in the human brain, facilitating the determination of epileptic foci of ADNFLE [76,77].

## 4. Recent Advancements in Diagnostic Techniques

The recent advancement in diagnostic strategies using various tools has been summarized in Table 1.

### 4.1. High-Resolution MRI (HRMRI)

HRMRI is a very sensitive and specific form of MRI. Image quality on MRI depends on many factors such as the thickness of the slice, the viewing angle, signal-to-noise ratio, matrix size, magnetic field strength, etc. [78]. The operational definition of HRMRI is restricted to MR attainments using clinically relevant 1.5–3.0 Tesla magnetic field strengths battered to intracranial arterial pathology. This technique can separate the arterial wall from the lumen of the proximal circle of Willis vessels. HRMRI can be performed by applying a 1.5 T magnetic field and restraining the field of view to focus on the point of concern. However, a higher field intensity at 3T has many benefits over orthodox 1.5 T MRI. The advantages of 3 T MRI involve faster image acquirement [79], increased signal-to-noise ratio [80], increased contrast-to-noise ratios, and better image quality [81,82].

Furthermore, this method is used in black-blood imaging, where the improved signal and distinction provided by 3 T, improves the detection of various complex blood flow abnormalities in the brain and can identify plaques in bigger arteries [79]. The 2D HRMRI is time-consuming and must be conducted by a neuroradiologist, who will ensure that enough sampling of the lesions of interest is obtained. High-resolution T1 image inversion recovery (IR) and high-resolution T2 image fluid-attenuated inversion recovery (FLAIR) is used [83].

### 4.2. Diffusion Tensor Imaging (DTI)

DTI is a special MRI technique that uses anisotropic diffusion to estimate the organization of white matter. Fiber tractography (FT) is a 3D reconstruction technique that uses the DTI data and assesses neuronal tracts. The DTI details provide information regarding the direction of water distribution in each voxel and can be used to measure the alignment of areas with white matter. Based on these data, it is possible to locate large, myelinated tracts, which provide additional surgical information. It can be used to detect optic radiation and predict visual field defects after surgery [84]. In addition, DTI may contribute to the acquisition of other numerical data, such as partial anisotropy, diffusivity, and connectivity indices, which could suggest subtle lesions of white matter, aiding in epilepsy diagnosis.

### 4.3. Magnetic Resonance Spectroscopy (MRS)

Proton-MRS evaluates neuronal robustness by measuring the peak of N-acetyl aspartate (NAA), a neuronal indicator, employed generally by contrasting its concentration with choline and creatine crests. The whole brain is not involved in conventional MRS measurements, unlike MRI, PET, and SPECT. Currently, only a few large voxels have been sampled by the proton MRS [85,86]. The comparatively poor signal-to-noise ratio of MRS and the relatively long time required to detect spectra make this technique slow. Localized EEG assessment and surgery results have demonstrated a reduction in the intensity of NAA. This, indeed, may prove helpful in localizing the epileptogenic foci in mTLE patients [87].

### 4.4. Magnetoencephalography (MEG)

Magnetoencephalography (MEG) is a process by which neurophysiological changes could be recorded. It is mainly used in human studies for systems-level brain function. The first MEG implemented brain activity recording took place over 40 years ago [88]. However, the refinement of the field has dramatically increased over the past decade [88]. MEG is a non-invasive high-resolution imaging technique that does not rely on direct neurophysiological measurements. It is the only practical brain tomography technique to offer both (a) high spatial (2–5 mm) and (b) temporal resolution (<1 ms) [85]. Another exclusive advantage of MEG over EEG is that there is no need for a reference electrode, which simplifies interpretation and network modeling along with connection analysis.

**Table 1:** Advanced diagnostic techniques for epilepsy.

Name of Imaging Technique	Study Population/Disease Model	Indication	Location/Tissue Examined	Effect in Epilepsy	AUC	Ref.
T1 and T2 weighted MRI	4-AP-induced seizures in rats	Diagnostic biomarker of epilepsy	Brain	In the cerebral cortex, hippocampus, amygdala, and medial thalamus, T2 relaxation time changes (recovered completely after 3 days)	-------	[3]
TSPO-PET	Kainic acid-induced SE in rats	Predictive biomarker of SE and SRS	Brain	Unregulated TSPO expression 14 days post-SE predicts SRS frequency and comorbidities associated with chronic SE	-------	[52]
iECEEG scalp EEG	Epileptic foci vs. other brain areas in humans assessed during epilepsy surgery	Diagnostic biomarker to identify epileptic foci	Brain	Existence of HFOs Conventional HFOs with waveform similarity Spikes	--------	[89]
fMRI	Patients with IGE vs. healthy controls	Diagnostic and predictive biomarker of IGE	Brain	Reduced DNM functional connectivity between anterior and posterior cortical seeds, seizure duration is positively associated with RSFC between Para hippocampal gyri and the PCC and negatively associated with connectivity between PCC and frontal lobe	---------	[90]
TARC/sICAM5 ratio assisted with video-EEG monitoring	Patients with focal epilepsy	Diagnostic biomarker for drug-resistant focal epilepsy	Blood/plasma	Increased TARC/sICAM5 ratio of these two proteins serves a vital role in epileptogenesis, as one is inducer of inflammation and the other inhibits it.	1.000	[91]
PET	Kainic acid-induced SE in rats	Diagnostic biomarker of SE	Brain	Decreased GABA receptor density and affinity in the hippocampus	---------	[92]
MRI	Hyperthermia-induced SE rats, with or without epilepsy	Diagnostic biomarker of epilepsy or prognostic biomarker of epilepsy development in hyperthermia-induced SE rats	Brain tissue regions– amygdala, thalamus	T2 relaxation time decreased in basolateral and medial amygdala	0.910 (Basolateral amygdala) 0.820 (Medial amygdala)	[93]
FDG-PET	Pilocarpine-induced SE	Diagnostic biomarker of SE	Brain	Decreased glucose metabolism, along with decreased brain connectivity	---------	[94]
PET	Pilocarpine-induced SE in rats	Diagnostic biomarker of SE	Brain	Decrease in global mGluR5 metabotropic glutamate receptor. Decreased focal in amygdala and hippocampus during chronic SE	--------	[95]
MRI	PTZ-induced seizures test in seizure susceptible or non-susceptible rats after TBI induction	Diagnostic biomarker of augmented seizure susceptibility after TBI	Brain tissue–cortex, hippocampus, thalamus	Decreased T2 relaxation time in medial thalamus	0.780	[96]
				Appearance of T1σ in S1 cortex, S1 HC, and Prh cortex	0.881 (S1 cortex) 0.857 (S1 HC) 0.929 (Prh cortex)	
				Appearance of T2 in the thalamus	0.893	
DTI-MRI	Patients with h benign vs. refractory mTLE (age and sex-harmonized)	Diagnostic biomarker for drug-resistant mTLE	Brain tissue–temporal lobe gray and white matter	Increased ipsilateral MD	0.670	[97]
				Decreased ipsilateral FA	0.770	
				Decreased ipsilateral HC volume	0.670	
FDG-PET	Pilocarpine-induced TLE	Prognostic biomarker of TLE	Brain	Decreased glucose metabolism in the hippocampus at the latent phase of the disease and neuronal loss	-------	[98]
DTI/DWI-MRI	Right and left TLE patients vs. healthy individuals	Diagnostic biomarker for TLE	Brain tissue–anterior corpus callosum	Reduced local diffusion homogeneity	RmTLE 0.935 LmTLE 0.919	[99]
MRS	Kainic acid-induced SE and amygdala kindling in rats	Biomarker of epilepsy	Brain	Sodium selenate prevents changes in mIns, NAA levels, volumetric changes, and FA	---------	[100]
T1 and T2 weighted MRI	Lithium–pilocarpine-induced SE in rats	Diagnostic biomarker of SE	Brain	T2 in the amygdala after 30 days of SE induction, strongly correlated with hyperactivity in the novel open field	--------	[101]
T1 and T2 weighted MRI	Kainic acid-induced MTLE in mouse	Diagnostic biomarker of MTLE	Brain	Hippocampal paroxysmal discharges (number and duration) are associated with T2 relaxation time	--------	[102]
Gadolinium-MRI	Patients with epilepsy vs. without epilepsy after TBI	Diagnostic biomarker for PTE in patients with TBI	Cerebral cortex	Area of gadolinium leakage around cortical lesion after TBI	0.850	[103]
Intracortical EEG	Rats having epilepsy vs. rats deprived of epilepsy after lateral fluid-percussion-induced TBI	Diagnostic biomarker for PTE in rats after lateral fluid-percussion induced TBI	Brain	Incidence of perilesional pHFOs and rHFOSs throughout the first 2 post-TBI weeks only in rats that will grow PTE	---------	[104]
MRS	Pilocarpine-induced SE -P21 rats which develop epilepsy vs. P21 rats which does not develop epilepsy	Diagnostic and prognostic biomarker for epilepsy and SE	Septal pole of the hippocampus	Augmented hippocampal mIns/tCr after 72 days of SE induction	0.830	[105]
MRS	Pilocarpine-induced SE in rats	Biomarker of SE	Brain	Increased expression of mIns post SE induction	-------	[105]
Combination of EEG and fMRI	Kainic acid-induced SE in Rhesus Monkey	Diagnostic biomarker of SE	Brain	Functional brain network disruption in chronic SE	------	[106]
MRI	Rats with or deprived of epilepsy after paraoxan-induced SE	Diagnostic and prognostic biomarker for epilepsy and SE	BBB pathology in the piriform network	Amplified T2- weighted signal	0.720	[107]
HMGB1- acetylated	65 patients with drug-resistant epilepsy and electrically induced rat SE model	Diagnostic biomarker for drug refractoriness	plasma	Acts on RAGE and TLR and increased expression in epilepsy	1	[108]
HMGB1- total	65 patients with drug-resistant epilepsy and electrically induced rat SE model	Diagnostic biomarker for epileptogenesis	plasma	Acts on RAGE and TLR and increased expression in epilepsy	1	[108]
HMGB1- bisulfide	65 patients with drug-resistant epilepsy and electrically induced rat SE model	Prognostic biomarker for SE	plasma	Acts on RAGE and TLR and increased expression in epilepsy	1	[108]
Diffusion MRI	Kainic acid and pilocarpine-induced SE in rats	Diagnostic biomarker of SE	Brain	Longitudinal changes in hippocampal diffusion due to astrocyte processes	-------	[109]
Diffusion MRI	Spontaneous recurrent seizures in cats	Diagnostic biomarker of epilepsy	Brain	Microstructural changes and hypoperfusion in the hippocampus and parietal cortex during ictal periods in cats	-------	[110]
Sleep EEG	Rats having epilepsy vs. rats deprived of epilepsy after lateral fluid-percussion-induced TBI	Diagnostic biomarker for PTE in rats after lateral fluid-percussion-induced TBI	Brain	Decrease of the extend of sleep occurrence of spindles at conversion from N3 to REM	0.907	[111]
Electrophysiology Interictal VEP-visual hyper excitability assessed using contrast response function	Patients having idiopathic generalized epilepsy vs. healthy individuals	Diagnostic biomarker for idiopathic generalized epilepsy	Brain	Relative lack of gain control at high contrasts	0.870	[112]
fMRI	17 patients with drug-resistant TLE with good post-operative seizure control vs. healthy controls	Diagnostic biomarker of TLE	Brain	fALFF reduction in ipsilateral amygdala	-------	[113]
Diffusion MRI	Spontaneous recurrent seizures in cats	Diagnostic biomarker of epilepsy	Brain	Decreased postictal hippocampal perfusion compared to ictal state	-------	[114]
TSPO-PET	Electrically induced SE in rats	Prognostic biomarker of SE	Brain	Unregulated TSPO expression up to 10 weeks after SE induction	-------	[115]
Theta dynamics in EEG	Rats with or without epilepsy after photo thrombotic stroke,Rats with or without epilepsy after bilateral hippocampal electrical stimulation-induced SE	Diagnostic biomarker of epilepsy or prognostic biomarker of epilepsy development in rodents having a brain injury	Brain	Absolute slope value of dynamic change in theta band	0.910	[116]
PET	Pilocarpine-induced SE in rats	Prognostic and diagnostic biomarker of SE	Brain	48 h after SE induction, the permeability of BBB gets increased in the hippocampus, piriform cortex, thalamus, and amygdala	------	[117]
EEG	Patients having acute anterior circulation ischemic stroke, which developed into epilepsy vs. not developed into epilepsy	Diagnostic biomarker of epilepsy or prognostic biomarker of acute anterior circulation ischemic stroke developing into epilepsy	Brain	Background asymmetry, Interictal epileptiform activity	0.810	[118]
EEG	Two cohort studies with- Patients having idiopathic generalized epilepsy and generalized spike-wave discharges on EEG, who are drug-resistant vs. drug-responsive	Diagnostic biomarker of drug resistance in idiopathic generalized epilepsy	Brain	Appearance of generalized polyspikes (burst of generalized rhythmic spikes lasting less than 1 s) in EEG during sleep	------	[119]
MEG coupled with behavioral evaluation	Human and animal models TLE	Early diagnostic biomarker of TLE	Brain	Coherence and alteration of theta and gamma rhythms	--------	[120]
EEG	Three different rodent models of epilepsy	Diagnostic and prognostic biomarker of epilepsy	Brain	Decrease in the non-linear dynamics dimension in EEG	≥0.886 in different models	[121]
EEG	A child with type 1 RCDP	Diagnostic biomarker of impending epilepsy	Brain	Transition from normal background to the appearance of focal epileptiform abnormality	-------	[122]
Interictal scalp EEG	22 children having CSWS	Predictive biomarker of seizures and cognitive outcome in CSWS	Brain	Presence of interictal HFOs around 80–250 Hz range (ripple band) and they are negatively associated with average IQ	-------	[123]
intracranial and scalp EEG	11 patients (6 M, 5 F; age range 21–41 years)	Diagnostic biomarker of postictal generalized EEG suppression	Brain	Delta- gamma phase-amplitude coupling (gradual decrease of phase-frequency in the coupling between delta, 0.5–4 Hz and gamma, 30+ Hz, followed by an increased coupling between the phase of 0.5–1.5 Hz signal and amplitude of 30–50 Hz signal)	-------	[124]
Scalp EEG	30 patients, suspected to have infantile spasms	Objective biomarker for active epileptic spasm	Brain	Increased HFO rates and coupling were identified between HFOs and SWA.	0.80–0.98	[125]
iEEG	11 patients having TLE (6 M, 5 F)	Diagnostic biomarker of SOZ	Brain	High amplitude of HFOs was observed in SOZs, and measuring the amplitude of HFOs serves a greater advantage over measuring the rate of HFOs, in the case of SOZ identification	0.948–0.960	[126]
MRS	35 patients with IGE (avg. age-32) vs. 35 healthy individuals (avg. age-31)	Diagnostic biomarker of IGE	Brain	Upregulated Cr expression in left thalamus (no difference in right thalamus). Downregulated NAA expression in right and left thalamus. Downregulated NAA/Cr ration in right and left thalamus.	-------	[127]
^18^F^-^FDG-PET/rs-fMRI	Patients with mTLE-HS vs. healthy individuals	Biomarker of epilepsy surgery in patients having mTLE-HS	Bain	Positive correlation between SUVR and rs-fMRI metrics, spatial correlation between SUVR and fMRI across the gray matter, and Higher fALFF/SUVR couplings, suggested altered bioenergetic coupling across gray matter and it was also found to be associated with seizure outcome.	------	[128]
[^11^ C] UCB-J PET	12 patients having TLE vs. 12 healthy controls	Diagnostic and predictive biomarker of TLE		Reduced [^11^ C] UCB-J binding in the seizure onset zone of patients having TLE		[129]
MRS	Kainic acid-induced MTLE in mouse (KA-MTLE model)	Biomarker of epileptic zone in MTLE	Brain	Upregulated GABA expression in the epileptic zone of mouse	------	[130]
iEEG	27 patients with SOZ in different parts of the hemisphere	Predictive biomarker of seizures	Brain	Temporal trends in HFO rates can predict preictal state and can differentiate between preictal and interictal periods for a few patients.	0.80	[131]

AUC: area under the curve; RCDP: rhizomelic chondrodysplasia punctata; HFO: high-frequency oscillations; SWA: slow-wave activity; iEEG: intracranial electroencephalogram; SOZ: seizure onset zone; CSWS: continuous spike-and-wave during sleep; fALFF: fractional amplitude of low-frequency fluctuations; IGE: idiopathic generalized epilepsy; DNM: default mode network; RSFC: resting-state functional connectivity; PCC: posterior cingulate cortex; 4-AP: 4-aminopyridine; SRS: spontaneous recurrent seizures; Cr: creatine; NAA: N-acetyl aspartate; mTLE-HS: medial temporal lobe epilepsy patients with hippocampal sclerosis; rs-fMRI: resting-state functional MRI; SUVR: ^18^ F-FDG standardized uptake value ratio; fALFF: fractional amplitude of low-frequency fluctuations.

The physical signal measured in MEG consists of the added magnetic fields generated by ionic currents inside the working neuronal zones, just like an electrical stream passing through a wire that produces magnetic fields around it. Currents inside the brain tissue, produced by ion flow, also generate magnetic fields corresponding to the same physical principles [132]. These magnetic fields in the neurons are smaller (10–15 T) in nature than the other magnetic fields that occur naturally, such as Earth’s magnetic field, which is about 10–5 T. Therefore, MEG recording is usually performed in magnetic-protected rooms using unique sensors and noise suppression software [133]. According to the neurological basis of the currents, MEG is mainly sensitive to dendritic currents in the pyramidal neurons of the neocortex [134]. Other cortical neurons in the brain also play a role in the signal, such as neural cells found in the subcortical and cerebellar regions [133]. The alignment depth and inherent synchronization of neuronal population also impacts the strength of MEG signals, although the most advanced high-density MEG systems are not affected by them [135].

In MEG, signal quantification occurs at the sensor level using typical systems with many sensors arranged in a helmet-like orientation. Therefore, a transformation of the sensor/channel measurements to source space (anatomical coordinates) are needed, generally achieved by using head models and optimized algorithms and related source estimation methods. This process is often termed MEG source modernization. Six types of waves are obtained: Delta (1–4 Hz), Theta (4–7 Hz), Alpha (8–14 Hz), Beta (14–26 Hz), Gamma (30–50 Hz), and High gamma (>50 Hz).

After performing MEG, a map of the magnetic activity of the brain is obtained. In the case of epilepsy, this magnetic map is distorted. By finding the location of the magnetic abnormality, the epileptogenic foci are determined. MES is used in the diagnosis of ADHD, PTSD, ASD, HIV-associated neurocognitive disorders (HAND), and epilepsy [88].

### 4.5. Quantitative Analysis of PET and MRI

PET is a visual assessment tool, but it can also be used for quantitative analysis to find the hypometabolic patterns in the brain as well as to quantify the degree of hypometabolism. In the past, there were concerns regarding the reliability of Quantitative PET (Q-PET) analysis, but at present, there is substantial evidence in the literature to support its reliability.

Q-PET analysis revealed that, during refractory temporal lobe epilepsy (rTLE), the amount of glucose metabolism gets decreased mainly in the inferior lateral temporal, inferior mesial temporal, and inferior frontal areas, as well as the thalamus areas, of the brain. Interestingly, after surgical treatment, glucose metabolism gets markedly increased in these regions [136]. A study demonstrated that FDG-PET and FMZ-PET were able to identify SOZs by quantifying the reduced uptake/binding of FDG and FMZ radiopharmaceuticals. Moreover, the results matched 86% and 71% with the stereo-electro-encephalography (SEEG)-obtained data, which is considered the gold standard for localizing the seizure onset zone (SOZ) in the epileptic brain [137]. In another study, Q-PET analysis revealed that patients with juvenile myoclonic epilepsy (JME) showed reduced dopamine uptake due to hindered dopamine transporter activities [138]. Epileptic patients suffering from hippocampal sclerosis (HS) are difficult to treat. Therefore, early, and accurate detection of HS is essential for providing proper care. Another study showed that Q-PET analysis of hippocampal volume or glucose uptake can increase the detection of HS [139]. Thus, Q-PET analysis can be a very useful tool to identify HS in epileptic patients.

Semiquantitative analysis by using standardized uptake value is the most accurate measure of hypometabolic areas, since quantitative analysis is associated with interobserver and intraobserver variability [140]. Semi-quantitative brain FDG-PET analysis was shown to increase the sensitivity of the diagnosis of autoimmune encephalitis (AE). Thus, it can be used in the future as a diagnostic tool for the early detection of AE [141].

Furthermore, some other studies integrated Q-PET analysis with other neuroimaging techniques to provide an accurate and well-quantifiable result. The MRI and PET techniques were merged to identify focal cortical dysplasias (FCDs). The combined use of MRI and PET was shown to increase the accuracy and demolish the false-positive results in this experiment [142]. Additionally, Q-PET analysis showed that, in the case of mesial temporal lobe epilepsy with HS (mTLE-HS), there is a decreased expression of benzodiazepine receptor density in the brain [143]. In this case, the findings were further confirmed with SPECT and MRI imaging to get an accurate result. Another study using the combination of MRI and Q-PET analysis revealed the hypometabolic regions in the brain of patients with non-lesioned extratemporal lobe epilepsy (ETLE) and mapped the hypermetabolic areas. These hypermetabolic areas were associated with a frequently spiking cortex and were rarely acknowledged in clinical readings. As these hypermetabolic areas may also present an epileptic focus, their detection can be helpful in the proper treatment of patients with ETLE [144].

### 4.6. EEG Analyses Methods

EEG analysis is a mathematical-based exploratory signal analysis, with software-based technologies being employed to extract the data from an electroencephalograph. The main prospect of these methods is to ease clinicians and basic researchers for gaining better knowledge of the brain and to assist in diagnosis and treatment choices. EEG analytical methods can be divided into five types as follows: first, the “*frequency domain analysis”* (spectral analysis) is one of the most convenient and powerful EEG analysis methods. It extorts the information from the frequency domain of EEG using Fourier transform and various statistical methods [145]. Another often-used frequency-domain method is “Power spectral analysis”. This method depicts the dispersal of signal power onto frequency [146]. The “Fast Fourier transform method” is another type of frequency-based analyses method where the characterization of obtained EEG waves is done by power spectral density estimation for the selective representation of EEG sample signals [147].

In the *Time domain analysis*, the analysis is based on time factor; however, it can also be obtained from the power spectrum. Thus, this method builds a link between time-based interpretation and conventional spectral interpretation [148]. In addition, it provides a way to measure the basic properties of signals online, using time-based calculations, which require less sophisticated equipment compared to classical frequency analysis [148]. This analysis is based on two aspects “Linear Prediction” and “Component Analysis”. Linear prediction is based on the estimated value equivalent to past outcomes, while component analysis is an unsupervised method in which the data set is mapped to a feature set [149].

The *time-frequency domain method* is a method by which the extraction and representation of transient biological signals can be achieved in two ways—wavelet transform and Hilbert–Huang transform. In wavelet transform, transitory factors can be precisely recorded (frequency and time context) through the wavelet decomposition of EEG records [150]. It is like a mathematical microscope that can analyze different scales of neuronal patterns and then investigate the slightest degeneration of brain patterns, ignoring other scales of collision [150]. It provides a more adaptable approach for the representation of the time-frequency of a signal for analysis. In wavelet transforms, long time frames are used to provide a finer low-frequency resolution and short time frames for high-frequency information [151]. Generally, wavelet transform can be in a continuous or discrete format [152]. On the other hand, Hilbert–Huang transform decomposes the EEG signals in different sets of oscillatory components, mainly Intrinsic Mode Function for recording instantaneous frequency data [153,154]. “Smooth pseudo-Wigner-Ville (SPWV)” distribution is another modified time frequency domain technique that includes “flattening by independent windows in time and frequency, namely, W w(τ) and W t(t)” [154].

*Non-linear methods*, non-linearity, and non-stationarity exist everywhere in nature, with EEG waves being no exception. The existence of non-linearity results in the interpretation of EEG waveforms as complex, and it also limits the application of linear methods. The interpretation of non-linear EEG patterns depends on the theory of non-linear dynamic systems (chaos theory). Several parameters are included for the analysis of non-linear waveforms viz., lyapunov exponent, correlation dimension, and entropies [155].

*Artificial neural networks* (ANNs), an analytical technique composed of a computerized neuronal network that is persuaded by the neuronal networks in the brain to segregate EEG data. Before putting the EEG data into the ANN, wavelet transform is mandatory [156]. Recurrent neural networks (RNNs) were used in the studies involving ANN implementations in EEG analysis. However, now, after the emergence of deep learning, it has been largely replaced by convolutional neural networks (CNNs). CNN is now the preferred method in EEG analysis employing deep learning. CNN is a special class of ANN that is mostly applied in the analysis of visual imagery. This method is mainly based on “the shared-weight architecture of the convolution kernels or filters that slide along the input features and provide translation equivariant responses known as feature maps” [157]. Deep CNN has proven to be a superior decoding choice since the cropped training deep CNN has reached competitive accuracies on the data sets [158]. Data entry and processing in the ANN compile large amount of data, and require high configuration data processing units for real-time processing. To overcome this challenge, cloud-based deep learning procedures may be utilized for hefty EEG data [159].

### 4.7. Other Advances in Data Analytical Techniques

CURRY analysis: This is a specialized technique that integrates the results of MRI and EEG. In this technique, the data obtained from the EEG are projected into the MRI in such a way that the detailed coordination of structural and functional data can be accomplished. By using this method, the location of a seizure can be easily determined.

Statistical parametric mapping (SPM): In this method, different parts of the brain with increased metabolic activity are compared, facilitating the determination of epileptic foci.

SISCOM: The Subtraction ictal SPECT co-registered to MRI (SISCOM) was first created with “Analyse” at the Mayo Foundation. The basic function of SISCOM is to equate the patient ictal scan with an interictal scan to create a subtraction image that is later inserted into and visualized on the patient’s MRI [160,161,162]. To nullify the dose variation, both ictal and interictal scans are normalized based on the intensity. In this way, individual data are co-registered collectively using a set method, and data subtraction can be further conducted. The resulting images are co-registered to MRI. Usually, 2 standard deviations are applied, even though some studies demonstrate better results with 1.5 SD [163]. Final brain maps obtained by SISCOM are known as perfusion maps, which depict areas of local hyper perfusion, indicating enhanced neuronal action in parts of the brain that are involved in seizure activity [163]. This is helpful in identifying the seizure onset zones in FCDs [164] and extra-temporal epilepsy [165].

STATISCOM: SISCOM has been proved to be a treasured data tool in epilepsy surgery, but its algorithm does not contain the physiologic inconsistency evaluation. STATISCOM involves a statistical ictal SPECT co-registered to MRI. The method is similar to SISCOM, but includes an additional step of data normalization by contrasting it with a control group using a statistical parametric mapping (SPM) analysis [166]. Ictal SPECT recognizes a single explicit region of seizure onset in 71% of mesial temporal and 83% of neocortical epilepsy case diagnoses [167]. STATISCOM is better than SISCOM for the identification of seizure foci before TLE surgery [166].

## 5. Limitations of Neuroimaging Techniques

Neuroimaging is one of the most advanced techniques of the healthcare sector. Daily technological advancements are making it more acceptable and devoid of error. However, neuroimaging also comes with some shortcomings and limitations which mainly involve. certain conditions under which the imaging techniques cannot be performed. These include the presence of a pacemaker, otic implant, aneurysm clip, metal in the eye, claustrophobia, implanted defibrillator, pregnancy, obesity, stents, and coils. The presence of any one of the above-mentioned factors can lead to false results; thus, before opting for neuroimaging techniques as a diagnostic tool, these factors are verified. Patient co-operation is also necessary in the case of neuroimaging [168]. If a patient is non-co-operative, the proper conduction of neuroimaging is not possible, since the altered emotional state of the patient can lead to false results. Inter-individual differences in the biochemical composition, processing of various life events, and basic emotions are probably co-responsible for inconsistent findings across different studies [169,170]. Therefore, before subjecting a patient to neuroimaging, their physio-psychiatric state is necessary to be evaluated, in order to avoid variation and false results.

Various artifacts can also affect the quality of the image produced. Aliasing or “wraparound” artifact occurs when the body part to be examined is larger than the field of view and the excess part can get projected in the other side of the image, which leads to masking of the underlying pathology. Motion artifact can also take place from arterial pulsation, CSF pulsation, patient movement, and respiration. Patient movement may occur even due to simple coughing or eye movement. Ferromagnetic substances present on the body of the patients, pillows, sheets, and in the air can lead to distortion of the image and can also conceal the areas of localized interest. Thus, the removal of various ferromagnetic substances from the experimental setup is necessary to avoid any kind of variation and false results [171,172,173].

Operator errors are also the most common and significant errors in neuroimaging techniques. Poor quality or a poorly ordered study could yield negative results, but the pathology may still exist and remain “image-able.” Common operator-induced errors include the lack of fat suppression for orbital MRI, lack of gadolinium administration, or improper voltage supply, misplacement of the detection sensors, false localization, and improper calibration of the instruments, affecting the ultimate result obtained. Therefore, an experienced and cautious experimenter is required for instrument operation, in order to minimize operator errors. Furthermore, nowadays, a few computer-guided lead placement instruments have also been developed to minimize the false localization and sensor placement errors.

Proper interpretation of the obtained results is also equally important, requiring a well-trained and experienced radiologist. Proper clinical localization or medical history records are also required to enable the radiologist to avoid overlooking any intracranial or intraorbital pathologies. Furthermore, there are some limitations in the neuroimaging techniques, which sometimes may not be able to differentiate between pathological processes. For instance, patients with a history of CNS malignancies can develop a focal neurologic change, such as homonymous hemianopia, after radiotherapy. Moreover, neuroimaging techniques cannot properly distinguish between necrotic and tumor recurrence lesions because it is very difficult for a radiologist to identify the type of lesion [174,175]. More research and developments are required to overcome these shortcomings and make neuroimaging error-free.

## 6. Biomarkers Associated with Diagnosis of Epileptogenesis

The identification of reliable biomarkers is the most practical solution for developing an economically feasible diagnostic technique [176,177]. It will provide a suitable screening tool that can identify potential subjects most likely to develop epilepsy due to genetic and structural deformities and help in its reduction at an early stage [178]. The FDA-NIH Joint Leadership Council, in 2015, Developed the Biomarkers, Endpoints, and other Tools (BEST, 2016) for better understanding and use of biomarker terminology. According to BEST, “A biomarker is a characteristic that is measured as an indicator of normal biologic processes, pathogenic processes, or responses to an exposure or intervention, including therapeutic interventions. Biomarkers may have molecular, histologic, radiographic, and physiologic characteristics.” The BEST biomarkers are divided into six categories: (a) risk or susceptibility biomarkers, (b) diagnostic biomarkers, (c) monitoring biomarkers, (d) prognostic biomarkers, (e) predictive biomarkers, and (f) safety biomarkers.

Biomarkers in epilepsy can be broadly divided into two types, i.e., “prognostic” (indicates epilepsy after a brain insult) and “diagnostic” (indicates ongoing epileptogenesis at that time). These biomarkers can serve various objectives, including (a) the use of risk biomarkers for the identification of a given epilepsy syndrome, e.g., genetic biomarkers, (b) prognostic biomarkers to predict the likelihood of accruing epilepsy, e.g., at a 2-year timepoint after traumatic brain injury (TBI), and (c) diagnostic biomarkers to identify ongoing epileptogenesis, even without the precise timing that the earlier brain insult occurred [176]. Table 2 summarizes the various types of biomarkers associated with epilepsy.

### 6.1. miRNAs as Biomarkers of Epileptogenesis

miRNAs (micro RNAs) are a special kind of RNA, controlling the post-translational gene expressions of various genes. About 60% of all gene expressions are directly controlled by miRNAs [179]. A single miRNA can affect the expression of several genes in a single pathway or a single gene in multiple pathways [180]. As an example, genetic deletion of miR-128 in mice resulted in the upregulation of more than a thousand mRNA transcripts, amongst which 154 were its predicted targets [181]. The effect of miRNAs in humans with epilepsy was first studied in 2010, reporting the upregulation of miR-146 expression in patients with TLE and hippocampal sclerosis [182].

Early functional studies have revealed that miRNAs are linked to seizure development, neuroinflammation, and changes in neuronal microstructure. For example, miR-134, which regulates LIM domain kinase 1, plays a vital role in altering the number and volume of dendritic spines on excitatory neurons [183,184]. On the other hand, miR-146a, miR-221, and mir-222 control immune response through the targeting of IL-1β and cell adhesion molecules [182,185,186]. miRNAs can also control cell differentiation, proliferation, and migration, which play a critical role in the epileptogenic pathway [187].

**Figure 2:**
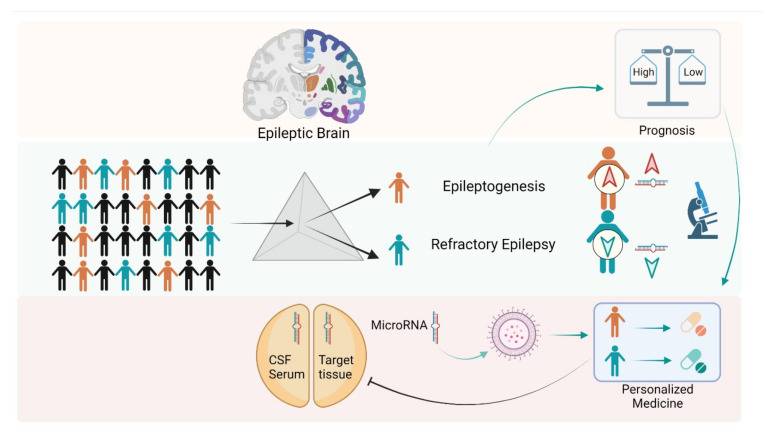
Role of microRNAs in diagnosis and personalized medicine in epilepsy. microRNAs (miRs) have been recognized as important tools for assessing the diagnosis of epileptic foci in patients. Changes in the expression levels of various circulatory/tissue specific miRs may differentiate epileptic phenomena such as epileptogenesis or drug responsive/drug-resistant epilepsy. Further, expression of a specific RNA may be altered using nucleic acid-based approaches (miR mimics, anti-miR, short hairpin RNA, small interfering RNA, aptamers, antisense oligonucleotide) for pursuing personalized medicine approaches to patients with epilepsy.

There is emerging evidence that miRNAs can serve as potential biomarkers of brain injuries, including epilepsy. A pool of brain-expressed micro RNAs may leak into the extracellular fluid from controlled exoplasm release or damage or even disruption of the BBB, allowing their passage into the blood. These miRNAs form a stable complex with blood proteins or get encapsulated in extracellular vesicles, remaining in the circulation for some time after their release [188]. Thus, a molecular biomarker of epilepsy is of great importance since it may enable diagnosis, assessing the risk of developing epilepsy, monitoring, and treatment (Figure 2).

Early animal studies suggested that a specific miRNA profile exists for different types of brain injuries, including epilepsy. A study has identified a set of circulating miRNAs, which includes the upregulated expression of miR-146 (a miRNA already linked with epileptogenesis) in blood [189]. In another study, a set of upregulated miRNAs’ expression was identified in the serum, which was observed to induce neuroinflammation, the dysregulation of protein synthesis, and neurodegeneration in epilepsy [190]. In that same study, two more miRNAs, miR-15a-5p and miR-194-5p, were identified to be downregulated in serum [190]. A set of downregulated miRNAs, including miR-301a-3p, miR-194p, miR-301a-3p, miR-30b-5p, and miR-4446-3p, were also detected in a different study serving as biomarkers of drug-resistant epilepsy [191]. Additionally, miR-323a-5p was found upregulated in serum as well as in the cerebral cortex of focal cortical dysplasia and drug-resistant epilepsy patients [187]. For temporal lobe epilepsy and mTLE-HS, several studies have identified a wide range of circulating miRNAs to be dysregulated, which can serve as a potential biomarker for epileptogenesis [192,193,194,195,196].

**Table 2:** MicroRNA-based diagnostic biomarkers for epilepsy.

Biomarker	Description	Role in Epilepsy	Indication	Specimen	Expression	Ref.
miR-34, miR-132, miR-134, miR-181a, miR-199a, miR-210	miRNAs associated with epilepsy play a vital role in epilepsy by targeting apoptosis, neuronal microstructure, transcriptional regulation, inhibitory neurotransmission, excitatory neurotransmission and by regulating transcription.	Antagomir of them reduces SE and protects the hippocampus	Diagnostic biomarker of SE and possible therapeutic targets of SE treatment	Serum, hippocampus	Increased	[187]
miR-128, miR-219, miR-23b, miR-124	miRNAs associated with epilepsy plays a vital role in epilepsy by targeting apoptosis, neuronal microstructure, transcriptional regulation, inhibitory neurotransmission, excitatory neurotransmission and by regulating transcription.	Agomir reduces SE and protects the hippocampus	Diagnostic biomarker of SE and possible therapeutic targets of SE treatment	Serum, hippocampus	Decreased	[187]
miR-134, miR-203	miRNAs associated with epilepsy plays a vital role in epilepsy by targeting apoptosis, neuronal microstructure, transcriptional regulation, inhibitory neurotransmission, excitatory neurotransmission and by regulating transcription.	Antagomir reduces SRS	Diagnostic biomarker and therapeutic target for SRS	Serum, hippocampus	Increased	[187]
miR-22	miRNAs associated with epilepsy plays a vital role in epilepsy by targeting apoptosis, neuronal microstructure, transcriptional regulation, inhibitory neurotransmission, excitatory neurotransmission and by regulating transcription.	Agomir reduces SRS	Diagnostic biomarker of SRS and possible therapeutic targets of SRS treatment	Serum, hippocampus	Increased	[187]
miR-106b-5p	A type of miRNA. MiR-106b-5p is an oncogene to attenuate the tumor suppressor CDKN1A	Inflammation, dysregulation of protein synthesis, and neurodegeneration	Diagnostic biomarker for epilepsy	Serum	Increased	[190]
miR-7d-5p	A type of miRNA, represses ER α expression	Inflammation, dysregulation of protein synthesis, and neuro degeneration	Diagnostic biomarker for epilepsy	Serum	Increased	[190]
miR-130a-3p	A type of miRNA targets HMGA1 regulation	Inflammation, dysregulation of protein synthesis and neuro degeneration	Diagnostic biomarker for epilepsy	Serum	Increased	[190]
miR-146a-5p	miRNA functions as a control switch between angiogenesis and cell death	Inflammation, dysregulation of protein synthesis and neuro degeneration	Diagnostic biomarker for epilepsy	Serum	Increased	[190]
miR-15a-5p	miRNA acts as a post-translational modifier of proto-oncogene MYB and can also target VEGF	Regulates: Fibrosis, inflammation, viability, and matrix degeneration	Diagnostic biomarker for epilepsy	Serum	Decreased	[190]
miR-194-5p	Tumour suppresser miRNA	Altered Nucleic and cytoplasmic functions, altered metal binding, and motif folding	Diagnostic biomarker for epilepsy	Serum	Decreased	[190]
miR-301a-3p	Supresses estrogen signaling, reduces the expression of ERα	Decreases the ESR1 mRNA and modulates inflammation	Diagnostic biomarker for drug-refractory epilepsy	Serum	Decreased	[191]
Combination of miR-194p, miR-301a-3p, miR-30b-5p, miR-4446-3p	These are various types of miRNAs affecting inflammation	Dysregulation of protein synthesis	Diagnostic biomarker for drug-refractory epilepsy	Serum	Decreased	[191]
miR-19b-3p	miRNA associated with epilepsy	Plays a role in epilepsy by inducing proteins that regulate apoptosis, tissue remodeling, gliosis, and neuroinflammation	Diagnostic biomarker for TLE	CSF	Decreased	[192]
miR-451a and miR-21-5p	miRNA associated with epilepsy	Plays a role in epilepsy by inducing proteins that regulate apoptosis, tissue remodeling, gliosis, and neuroinflammation	Diagnostic biomarker for SE	CSF	Increased	[192]
miR-134	miR-134 is a family of MicroRNA precursors found in mammals, including humans	Targets Lim kinase 1, a protein involved in dendritic spine dynamics 22 and doublecortin	Diagnostic biomarker for mTLE	plasma	Decreased	[193]
miR-27a-3p, miR-328-3p and miR-654-3p	miRNAs associated with epilepsy	Association with growth factor and apoptosis signaling (p53 pathway)	Diagnostic biomarker of TLE	Blood plasma	Increased	[194]
circ-EFCAB2	Circular RNAs, long noncoding RNAs, acts as templates and regulates transcription	Have a role in epilepsy as they regulate gene expression as microRNA sponges	Diagnostic biomarker and potential treatment target in TLE	Temporal cortices	Increased	[195]
circ-DROSHA	Circular RNAs, long noncoding RNAs, acts as templates and regulates transcription	Have a role in epilepsy as they regulate gene expression as microRNA sponges	Diagnostic biomarker and potential treatment target in TLE	Temporal cortices	Decreased	[195]
miR-145	miRNA associated with macrophage differentiation, phagocyte migration, proliferation	Reduced expression in the hippocampus of epileptic brain	Diagnostic biomarker of mTLE-HS	Blood, hippocampus	Decreased (hippocampus), increased (blood)	[196]
miR-181c	miRNA targets dopaminergic, serotonergic synapses, and has a role in BBB disruption and breast cancer cell metastasis	Overexpressed in the hippocampal region of mice after epileptic seizures	Diagnostic biomarker of mTLE-HS	Blood, hippocampus	Increased in hippocampus and blood	[196]
miR-199a	Especially expressed in the olfactory bulb, cortex, hippocampus, hypothalamus, brain stem, dorsal root ganglia	Over-expression in epileptic brain tissues	Diagnostic biomarker of mTLE-HS	Blood, hippocampus	Increased in hippocampus and blood	[196]
miR-1183	miRNA which was observed to lose its function in breast tumors, and it was observed to be downregulated in Kaposi sarcoma biopsies. Although, upregulation was observed in colorectal tumors and RHD.	It targets genes-CXCR4, EGF, and EGFR. Thus, it is considered a potential biomarker.	Diagnostic biomarker of mTLE-HS	Blood, hippocampus	Increased in hippocampus and blood	[196]
miR-106b miR-146a miR-301a	Epilepsy related miRNA	Regulation of TLR, IL-1, receptor associated kinases and TRAF6	Diagnostic biomarker for epilepsy	Serum	Increased	[197]
miR-194-5p, Combination of miR-106b and miR-146a	Epilepsy related miRNA	Inhibits cell proliferation	Diagnostic biomarker for epilepsy	Serum	Decreased	[197]
miR-129-2-3p	Non-invasive miRNA biomarker for epilepsy	Repress cell growth, colony formation and targets BCL_2_L_2_	Diagnostic biomarker for drug refractory TLE with FCD	Cortical tissue and plasma	Increased	[198]
miR-4521	Promising Ovel biomarkers	Nucleic and cytoplasmic functions altered, altered metal binding and motif folding	Diagnostic biomarker for FCD with refractory TLE	Cortical brain tissue, serum	Increased	[199]
MMP2, MMP3	MMP2, MMP3	Epileptic focus formation and stimulation of seizures	Diagnostic biomarker for Epilepsy	brain	Decreased	[200,201]
miR-30, miR-378, miR-106b and mir-15a	miRNA associated with epilepsy	Expression is positively associated with seizure frequency, however, miR-30 expression gradual declines with the progression of the disease and negatively affects the CAMK4 expression	Diagnostic and prognostic biomarker of epilepsy	Serum	Increased	[202]
miR-211	miRNA which shifts the threshold for spontaneous and pharmacologically induced seizures	Downregulation induces hyper synchronization and nonconvulsive, convulsive seizures along with alterations in cholinergic and TGFBR2 signaling pathways	Diagnostic biomarker of epilepsy	Forebrain	Decreased	[203]
miR-134	miRNA associated with epilepsy serves an important role in inter-neuronal signaling by targeting dendrites	Upregulation of miR-134 was found in the rodent models of SE.	Diagnostic biomarker of epilepsy	Plasma and CSF	Increased	[204]
miR-323a-5p	miRNA associated with epilepsy	Associated with duration of epilepsy and seizure frequency	Diagnostic biomarker of FCD and DRE	Cerebral cortex, blood plasma	Increased	[205]
miR-3613-5P miR-4668-5P miR-8071, miR-197-5P	miRNA associated with epilepsy	Inflammation in brain and neuronal tissues	Diagnostic biomarker for mTLE-HS	Plasma exosomes	Decreased	[206]
miR-146a and miR-106b	miRNA associated with epilepsy	Important regulators of the innate immune response in the modulation of astrocyte-mediated inflammation	Diagnostic biomarker of childhood epilepsy	Plasma	Increased	[207]
miR-106b	Belongs to the miR-17 family and are associated with CVDs and tumors	Highly expressed in the serum of epilepsy patients	Diagnostic and prognostic biomarker of epilepsy in children	Serum	Increased	[208]
miR-15a-5p	miRNA associated with epilepsy and acts as a regulator in endometrial cancer	Upregulation reduced the apoptosis and increases the cell viability of hippocampal neurons, which were dysregulated by TLE	Diagnostic biomarker of TLE in children	Serum	Decreased	[209]
miR-135b-5p	miRNA associated with epilepsy and cancer cell proliferation, migration.	Reduces post epileptic dysregulation of cell viability and apoptosis of hippocampal neurons by targeting SIRT1.	Diagnostic biomarker of TLE in children	Plasma	Decreased	[210]
miR-93-5p, miR-199a-3p and miR-574-3p	miRNA associated with epilepsy	Found to be dysregulated in epilepsy	Diagnostic biomarker of TLE	Plasma	Increased	[211]
miR194-2-5p, miR15a-5p, miR-132-3p, and miR-145-5p	miRNA associated with epilepsy	Plays important role in the pathogenesis of FCD and refractory epilepsy by regulating mTOR, P13K-Akt, P53, TGF- β signaling pathways, and cell cycle.	Diagnostic and prognostic biomarker for refractory epilepsy	Serum	Increased	[212]
miR-328-3p	miRNA associated with epilepsy	Important peripheral biomarker of epilepsy	Diagnostic biomarker of mTLE-HS	Serum	Increased	[213]
miR-654-3p	miRNA associated with epilepsy	Important peripheral biomarker of epilepsy with statistical power to differentiate between Engel I (good surgical prognosis) and Engel III-IV (unfavorable surgical prognosis) patients	surgical prognosis biomarker of MTLE-HS	Serum	Increased	[213]
miR-134 and miR-146a	miRNA associated with epilepsy, and they also had a role in neuroinflammation, dendritic functionality	Their upregulated expression indicates a higher risk of developing DRE, independent of temporal lobe sclerosis, epilepsy duration, familial history, age at first seizure, age, body mass index (BMI), smoking behavior, and gender	Predictive and prognostic biomarker of DRE	Serum	Increased	[214]
miR-182	miRNA associated with epilepsy, neuroinflammation, and apoptosis	Upregulation inhibited the expression of APLN, which serves a neuroprotective role in epilepsy. Moreover, it’s upregulation induces apoptosis.	Diagnostic biomarker of epilepsy	Hippocampal neurons	Increased	[215]
MiR-146a, miR-155 and miR-132	miRNA associated with epilepsy	Plays important role in neuroinflammation, neuroprotection, neurodegeneration, and neuronal growth, related to epilepsy	Diagnostic and prognostic biomarker of GGE	Serum	Increased	[216]
miR-194-5p	miRNA associated with epilepsy	Hyperexpression increases cell viability which was reduced by TLE. Targets the IGF1R gene directly and reduces apoptosis of hippocampal neurons.	Diagnostic biomarker and treatment target of TLE in children	Plasma	Decreased	[217]
miR-142	miRNA associated with epilepsy	Expression is associated with anti-inflammatory signaling in epileptogenic tubers from a tuberous sclerosis complex	Diagnostic biomarker of TLE and prognostic biomarker for drug-resistant TLE	Serum	Increased	[218]
miR-146a	miRNA associated with epilepsy	Diagnostic miRNA in genetically generalized epilepsy because of its role in propagating inflammation of the hippocampus	Diagnostic biomarker of TLE	Serum	Increased	[218]
miR-223	miRNA associated with epilepsy	Expression is associated with anti-inflammatory signaling in epileptogenic tubers from a tuberous sclerosis complex	Diagnostic biomarker of TLE and prognostic biomarker for drug-resistant TLE	Serum	Increased	[218]

TARC: thymus and activation-regulated chemokine; RAGE: receptor for advanced glycation end products; TLR: toll-like receptor; mTLE: mesial temporal lobe epilepsy; HS: hippocampal sclerosis; IGF1R: insulin-like growth factor 1 receptor; SIRT1: sirtuin (silent mating type information regulation 2 homologs) 1; FCD: focal cortical dysplasia; MTLE-HS: mesial temporal lobe epilepsy with hippocampal sclerosis; DRE: drug-resistant epilepsy; APLN: apelin; GGE: genetic generalized epilepsy; RHD: rheumatic heart disease; CAMK4: calcium/calmodulin-dependent protein kinase type IV; SRS: spontaneous recurrent seizures.

The evidence of miRNAs as a potential biomarker of epilepsy is largely preclinical, and clinical evidence with significant results is still missing. miRNAs have great potential to be served as a potential biomarker, but more research is required to solidify their application in real life.

### 6.2. Genetic Biomarkers

A few genetic biomarkers have been identified that indicate an increased risk of structural epileptogenesis. Two markers associated with post-stroke epilepsy are mutations in CD-40-1C/T or Rs671 genes [219].

### 6.3. Molecular Analysis

Two biomarkers have been identified in epileptogenesis diagnosis. Increased plasma levels of high-mobility group box 1 protein have been reported after unilateral hippocampal electrical stimulation-induced status epilepticus [108]. A 1.5-fold increase in cortisol levels in a 3 cm scalp hair sample was reported in a 6–12-year-old child with benign childhood epilepsy syndromes when the sample was analyzed within 24 h of the first seizure [220]. Generally, under normal physiological conditions, cortisol accumulation in hair takes several weeks [221,222,223]. Therefore, these studies suggest increased hair cortisol levels and dysfunction of the hypothalamic–pituitary–adrenal axis is a potent indicator of epileptogenesis.

### 6.4. Molecular Profiling after Surgery

It had been reported that there is a relative reduction in ZNF852, CDCP2, PRRT1, FLJ41170, and 7RNA probes in patients who become seizure-free, after lobectomy for interactable TLE patients [224]. An amplified hippocampal myoinositol/total creatine ratio is a potential diagnostic biomarker for epileptogenesis in the case of lithium–pilocarpine-induced status epilepticus [105]. Another potential biomarker of diagnosis of status epilepticus is upregulation of miR-451a or miR-21p, or downregulation of miR-19b in the CSF [192]. The SE unit involved patients with focal SE, nonconvulsive SE, and generalized tonic-clonic SE, although, after TBI or stroke, the diagnosis of nonconvulsive SE becomes difficult [192]. Risk biomarkers, including a CD1 background or carrying an APP/PS1 mutation, indicate a greater susceptibility towards epilepsy [225,226]. These groups need better surveillance and care.

## 7. Conclusions and Future Aspects

The diagnosis of epilepsy is quite complex and subjected to many variations in the patients or the experimental environment. Patient variations may occur on an individual population scale, such as drug intake, treatment protocols, physiological and pathological conditions leading to misdiagnosis, and several serious issues. For the effective treatment of epilepsy, the location of epileptic foci, as well as the spread to the other parts of the brain, should be properly identified, especially for drug-resistant cases. The EEG can help determine whether a patient suffers from epilepsy or not by detecting abnormal paroxysmal discharge patterns. Following the preliminary examination with EEG, the foci of epileptogenesis can be determined by various techniques, including MRI, PET, SPECT, and CT scans. The basis of these techniques is different, but they may provide high-quality images that significantly contribute to the facilitation of the diagnosis of epilepsy. Currently, the research is focused on the targeting of various receptors and transporters affected during epilepsy, such as 5-HTT and 5-HTR. This molecular imaging of the brain holds great potential in the future for better determination of epileptic foci as well as epileptogenesis. To achieve an accurate diagnosis of epilepsy, all findings must be incorporated into one image. Thus, producing a 3D image and video of the brain depicting the areas of epileptogenesis and the spread into the other parts of the brain could be substantially helpful. Some recent advances in this kind of integration are discussed in this review. Along with these, the development of novel biomarkers for the diagnosis of epileptogenesis is gaining considerable interest in the field of research. Before the application of neuroimaging instruments, the detection of potential biomarkers is a cheap and useful tool to confirm epilepsy before the implication of expensive procedures. The development of these techniques will significantly aid in the deep understanding of several changes occurring inside the brain as well as during and between seizures, eventually improving diagnosis and effective clinical management.

## Data Availability

No new data were created or analyzed in this study. Data sharing is not applicable to this article.

## Abbreviations

CT scan—Computed Tomography; MRI—Magnetic Resonance Imaging; PET—Position Emission Tomography; SPET—Single Photon Emission Tomography; EEG—Electroencephalography; MEG—Magnetoencephalography; MRS—Magnetic Resonance Spectroscopy; DTI—Diffusion Tensor Imaging; HRMRI—High-Resolution MRI
